# Pre-operative plasma VEGF-C levels portend recurrence in epithelial ovarian cancer patients and is a bankable prognostic marker even in the initial assessment of a patient

**DOI:** 10.1186/s13048-024-01398-0

**Published:** 2024-04-09

**Authors:** J. Bhaskari, Rahul Bhagat, V. Shilpa, C.S. Premalata, Lakshmi Krishnamoorthy

**Affiliations:** 1 Department of Biochemistry, Kidwai Memorial Institute of Oncology, Bangalore, Karnataka India; 2 Department of Pathology, Kidwai Memorial Institute of Oncology, Bangalore, Karnataka India; 3 Present Address: National Centre for Biological Sciences, GKVK campus, Bangalore, Karnataka India; 4 Present Address: Oncostem Diagnostic Pvt Ltd, Bangalore, Karnataka India; 5 Present Address: Department of Biochemistry, Sri Shankara Cancer Hospital and Research Centre, Bangalore, 560004 Karnataka India

**Keywords:** VEGF-C, COX-2, Ki-67, eNOS-nitric oxide, Epithelial ovarian cancers, Prognostic markers.

## Abstract

**Purpose:**

Our explorative study assessed a panel of molecules for their association with epithelial ovarian carcinomas and their prognostic implications. The panel included tissue expression of VEGF-C, COX-2, Ki-67 and eNOS alongside plasma levels of VEGF-C and nitric oxide.

**Methods:**

130 cases were enrolled in the study. Plasma levels were quantified by ELISA and tissue expressions were scored by immunohistochemistry. The Chi square and Fischer’s exact test were applied to examine the impact of markers on clinicopathological factors. Non-parametric Spearman’s rank correlation test was applied to define the association among test factors.

**Results:**

Plasma VEGF-C levels and COX-2 tissue expression strongly predicted recurrence and poor prognosis (< 0.001). Tissue Ki-67 was strongly indicative of late-stage disease (< 0.001). The aforementioned markers significantly associated with clinicopathological factors. Nuclear staining of VEGF-C was intriguing and was observed to correlate with high grade-stage malignancies, highly elevated plasma VEGF-C, and with recurrence. eNOS tissue expression showed no significant impact while nitric oxide associated positively with ascites levels. Tissue expression of VEGF-C did not associate significantly with poor prognosis although the expression was highly upregulated in most of the cases.

**Conclusion:**

Plasma VEGF-C holds immense promise as a prognostic marker and the nuclear staining of VEGF-C seems to have some significant implication in molecular carcinogenesis and is a novel finding that commands further robust scrutiny. We present a first such study that assesses a set of biomarkers for prognostic implications in clinical management of epithelial ovarian carcinomas in a pan-Indian (Asian) population.

**Supplementary Information:**

The online version contains supplementary material available at 10.1186/s13048-024-01398-0.

## Introduction

The stealth queen of gynaecologic maladies, Epithelial ovarian cancer (EOC) is not only lethal but also one of the most rampant afflictions of the female reproductive system, second only to endometrial cancer [1]. The incidence of EOCs vary globally, with the Caucasian race having the highest rate of incidence and the pacific south Asian race, the lowest. An apparent lower risk for coloured women has also been suggested. Its incidence is relatively low in India, although, there is a steady increase in the prevalence rate and is the leading cause of death from cancer in Indian women [2].

The lethality is inherent in its stealth at early stages, being mostly asymptomatic until late stage. Even in advanced stages, patients present with generic symptoms that can flummox medical practitioners, like abdominal discomfort, bloating, dyspepsia, early satiety, lack of appetite, malaise, urinary frequency and change in weight. Biopsies are referred to when a cancer diagnosis becomes imminent, and by then the cancer turns out to be too advanced for a successful treatment. Diagnostic tests such as plasma CA-125 levels and transvaginal ultrasound scanning hardly shed any light on the aggressiveness of the condition and lack any credible prognostic or predictive capacity.

The current mode of treatment typically involves surgical de-bulking followed with platinum-based chemotherapy which is only partially effective. Most patients eventually relapse and die of the disease post treatment with a median progression-free survival of 18 months [3]. An effective prognostic marker helps to stratify patients with relative ease and helps identify patients that are likely to have poor response to standard treatment, and hence requiring customised treatment for a better shot at disease-free survival. This can especially help avoiding unnecessary heavy treatment for patients with good prognosis, consequently reducing treatment associated morbidities in them. Age, stage, histology, grade, ascites volume, and residual disease are some of the currently interpreted prognostic factors in ovarian cancer [4, 5], most of which are invasive tests. Even with these, the morbidity and mortality associated with EOCs are so high that the need for more robust and efficient prognostic markers is quite irrefutable to enable a customised treatment for patients that can reduce treatment associated morbidities.

Neovascularization, a process of composite yet subtle modulation by several factors that tip the balance towards the formation of new blood vessels, is at the heart of cancer cell survival. The two major well explicated components of neovascularization are vasculogenesis and angiogenesis. While vasculogenesis is the process of new blood vessel formation from hemangioblasts, angiogenesis (and lymphangiogenesis) is the process of new blood vessel formation from pre-existing vasculature through capillary sprouting. Amongst the several factors that modulate neovascularization, the Vascular Endothelial Growth Factor (VEGF) family of proteins is the major robust player in maintaining vasculature, both physiologic and pathologic.

The VEGF family of proteins includes the VEGF-A, VEGF-B, VEGF-C, VEGF-D and PIGF. The tyrosine kinase receptor family includes the VEGFR-1, VEGFR-2 and VEGFR-3. The VEGFR-1 and R-2 are expressed in adult vascular endothelial cells that are transduced by VEGF-A and are primarily involved in angiogenesis. VEGFR-3 is expressed exclusively in the veins and lymphatic endothelial cells and are receptors to VEGF-C and VEGF-D that are primarily involved in lymphangiogenesis.

VEGF-C transduces downstream signalling through both VEGFR2 and VEGFR3. It is a prepro-protein that is subjected to proteolytic processing to generate the mature form of the growth factor. The unprocessed form binds to VEGFR-3 which is mainly expressed in the lymphatic vasculature and hence the mainstay to lymphangiogenesis. The fully processed form can bind to both VEGFR-2 and VEGFR-3 forming a heterodimer that can augment VEGFR-2 downstream signalling. Hamada K et al. demonstrated elaborate control of vasculogenesis and hematopoiesis through VEGFR-3 signalling. They showed that VEGF-C binding to VEGFR-2 worked synergistically with VEGF-A and binding of VEGF-C to VEGFR-3 had regulatory effects on VEGFR-2 signalling. In brief, they demonstrated that VEGF-C signalling through VEGFR-3 played an eminent role in vasculogenesis and that the levels of free VEGF-C could be critical for this signalling. They also established that VEGF-C signalling though VEGFR-2 enhanced VEGF-A activity [6]. Assessing VEGF-A expression in plasma, tissue and ascitic fluids in a preceding study, we had shown that VEGF-A levels significantly correlated with high grade malignances and as a reasonable marker for malignant EOCs [7, 8]. Although pathologic angiogenesis is intrinsic to carcinogenesis, lymphangiogenesis is undeniably important since they present an obvious means of distant metastasis leading to poor prognosis.

The study of VEGF-C tissue expression could be pivotal in carcinomas since they not only can augment lymphatic endothelial cell migration, proliferation, and survival (through VEGFR-3), but can also contribute the same in the vascular endothelium (through VEGFR2/R3 heterodimer). The hypoxic condition in solid tumours is the most important molecule that up-regulates multiple factors that cause an angiogenic “switch-on” by the binding of Hypoxia Inducible Factor 1- α (HIF1- α) to the Hypoxia responsive element (HREs) in the promoter of target genes. The stimulation of VEGF-C expression, unlike VEGF-A, is not influenced directly by HIF1-α since VEGF-C gene promoter does not contain a HRE sequence [9]. Instead, a HIF1- α independent translational up-regulation of VEGF-C expression has been shown under hypoxia [10]. Furthermore, other factors stimulated by HIF1- α under hypoxia work in tandem to modulate VEGF-C expression.

Recent studies have shown that VEGF-C upregulation in many malignant cell lines has been induced by the enzyme COX-2 [11]. COX-2, a rate-limiting enzyme that is involved in the metabolism of prostaglandins, is known to be highly expressed in malignancies and is also associated with poor prognosis. Many studies have shown a strong correlation between COX-2 and VEGF-C [12, 13]. Incriminating studies have been conducted in malignant cell lines, lung carcinomas, oesophageal carcinomas, and breast carcinomas. Over-expression of VEGF-C has been observed in several carcinomas, such as lung carcinomas, head and neck cancers, breast carcinomas, prostate carcinoma, colon carcinomas, gastric cancers, and papillary thyroid carcinomas [11–17].

A study on the impact of VEGF-C was warranted since VEGF-C not only works towards lymphangiogenesis but also impacts angiogenesis via COX-2 induction and the VEGFR2/R3 downstream signalling. VEGF-C is shown to enhance c-Jun binding to cAMP response element of the COX-2 promoter, thereby inducing it. The VEGF-C/VEGFR-3 downstream signalling induces COX-2 via the JNK/AP-1 pathway [18]. Studies have also shown that COX-2 induced further expression of VEGF-C in a positive feedback loop mechanism. Thus, the VEGF-C/COX-2 signalling nexus has been implicated for mutual modulatory action through a common pathway in several studies [19].

VEGF-A/R2 downstream signalling has been demonstrated to induce both COX-2 and eNOS to facilitate tissue permeability and cellular migration, thus enabling tumorigenesis. VEGF-A/R2 is known to modulate COX-2 and eNOS mediated nitric oxide levels, and thereby possibly induce VEGF-C expression, despite VEGF-C lacking a sensitivity to hypoxic induction. The study of the VEGF-C/COX nexus has not been explored in epithelial ovarian carcinomas (EOCs).

Exploring these cross-talks, we broadened the scope of our initial study on VEGF-A and ventured to explore the impact of the tissue expression of COX-2, VEGF-C, eNOS, Ki-67 (a proliferation marker) and the corresponding plasma levels of VEGF-C and NO. We have also examined the association of these factors with clinicopathologic factors of EOC and their prognostic implications. To the best of our knowledge, this is a first such study to present a cohesive data from a Pan-Indian demographic perspective on EOCs.

## Materials and methods

### Sample collection

A total of 130 patients who were diagnosed with primary epithelial ovarian tumours at the Kidwai Memorial Institute of Oncology, Bangalore, India, between 2011 and 2013 were enrolled into the study. The initial institutional diagnosis of epithelial ovarian cancer was confirmed by review of pathologic slides by senior pathologists. Of the 130 samples collected, 95 (73.1%) were malignant cases, 17 (13.1%) were borderline or Low Malignant Potential (LMP) tumours and 18 (13.8%) were benign cases. 15 healthy control subjects, matched for age and menopausal status formed the control group. The demographic details of the study cases are given in Table 1.

**Table 1:** Demography of case cohort

Characteristics	Malignant, n(%)	Borderline, n(%)	Benign, n(%)	Total	*P* value
Number (n)	95 (73.1)	17 (13.1)	18 (13.8)	130 (100)	
Age (mean ± SD)	51 ± 12	46 ± 16	50 ± 14	51 ± 12	**0. 3378**
Mensus status					**0.602**
Pre mensus	28 (29.5)	7 (41.2)	5 (27.8)	40 (30.8)	
Post mensus	67 (70.5)	10 (58.8)	13 (72.2)	90 (69.2)	
FIGO stage					**< 0.001**
I and II	36 (37.9)	17 (100)	---	53 (40.8)	
III and IV	59 (62.1)	0 (0)	---	59 (45.4)	
Grade					**< 0.001**
I and II	39 (41.1)	17 (100)	---	56 (50)	
III and UD	56 (58.9)	0 (0)	---	56 (50)	
Histopathology					**< 0.001**
Serous	57 (60)	8 (47.1)	9 (50)	74 (56.9)	
Mucinous	10 (10.5)	8 (47.1)	9 (50)	27 (20.8)	
Endometrioid	5 (5.3)	1 (5.9)	0 (0)	6 (4.6)	
Clear cell	5 (5.3)	0 (0)	0 (0)	5 (3.8)	
Poorly differentiated	18 (18.9)	0 (0)	0 (0)	18 (13.9)	
Bilateral affliction	59 (45.4)	5 (29.4)	0 (0)	64 (49.2)	ND*
Pre-Op CA125					**< 0.001**
0–35 U/mL	5 (5.3)	5 (29.4)	12 (66.7)	22 (16.9)	
35–110 in U/mL	7 (7.4)	6 (35.3)	5 (27.8)	18 (13.8)	
110–1000 U/mL	51 (53.7)	5 (29.4)	1 (5.5)	57 (43.9)	
> 1000 U/mL	32 (33.7)	1 (5.9)	0 (0)	33 (25.4)	
Ascites					**< 0.001**
Nil	17 (17.9)	6 (35.3)	14 (77.8)	37 (28.5)	
< 500 ml	25 (26.3)	10 (58.8)	4 (22.2)	39 (30)	
> 500 ml	53 (55.8)	1 (5.9)	0 (0)	54 (41.5)	
Residual disease					**< 0.001**
Nil	44 (46.3)	12 (70.6)	18 (100)	74 (56.9)	
< 1	27 (28.4)	2 (11.8)	0 (0)	29 (22.3)	
> 1	22 (23.2)	0 (0)	0 (0)	22 (16.9)	
Not known	2 (2.1)	3 (17.6)	0 (0)	5 (3.9)	

*ND – Not Determined; Nil – Nought (no ascites or no residual disease)

5 ml of blood was collected from all subjects in heparin vacutainers. The plasma was separated immediately by centrifugation and the cell pellet was stored at -20˚C for DNA isolation and genotyping. The separated plasma samples were stored at -80˚C until analysis for VEGF-C levels and NO levels. All samples were collected prior to treatment.

The tumours were graded according to WHO criteria and staged according to the Federation of Gynaecology and Obstetrics (FIGO) classification. Clinicopathological details such as FIGO stage, histological grade, subtype, pre-operative CA-125 levels, Ki-67 tissue expression, residual disease, recurrence (within a period of 3 years), and follow-up were abstracted from the patient’s medical records. Study approval was given by the Institutional Review Board and the Medical Ethics Committee of Kidwai Memorial Institute of Oncology and written informed consent was obtained from all participants.

### VEGF-C plasma level measurement

Plasma VEGF-C levels were measured by Enzyme Linked Immuno Sorbent Assay using Quantikine ® ELISA for Human VEGF-C (R&D Systems, Minneapolis USA) following manufacturer’s protocol. All samples and standards were analysed in duplicates.

Plasma Nitric oxide levels were measured by Enzyme Linked Immuno Sorbent Assay using Quantikine ® ELISA for Nitric oxide measurement (R&D Systems, Minneapolis USA) following manufacturer’s protocol. All samples and standards were analysed in duplicates. The total nitric oxide level was calculated as a total of the nitrite and nitrate moieties in the sample.

### Immunohistochemistry

IHC was performed on all 130 case samples and 15 samples of normal ovarian tissue as controls. Formalin-fixed paraffin-embedded tissues were used for constructing tissue microarray (TMA) blocks. Selected cancer foci were marked on H&E-stained sections. A tissue-arraying instrument (Beecher Instruments, Sun Prairie, WI, USA) was used to acquire cylindrical tissue cores with a diameter of 2 mm from histologically representative areas of the donor blocks. 36 tissue cores were composited into a single recipient paraffin block at defined array positions. 2 tissue cores were obtained from each specimen and represented in duplicates in the array in this study. 5 mm sections were cut from the TMA block, mounted on 2% aminopropyltriethoxysilane-coated glass slides, dewaxed in xylene twice for 15 min and rehydrated in a graded series of isopropanol (100, 90, 80, 70%). Steam antigen retrieval was performed for 20 min in Tris EDTA (pH 9.0). The sections were treated with 3% H_2_O_2_ for 20 min to block any endogenous peroxidase activity. Nonspecific binding sites were blocked with 2% skimmed milk for 30 min. The sections were then incubated with primary antibody for 1 h 30 min at room temperature, followed by broad spectrum secondary and tertiary antibody (Biogenex Laboratories Inc, Fremont, CA, USA) for 30 min each at room temperature. Colour development was performed using 3, 39 diaminobenzidine-hydrogen peroxide for 10 min. The sections were then counterstained with haematoxylin. Section with no primary antibody added was used as a negative control. Mouse anti human VEGF-C monoclonal antibody (Santa Cruz) was used at 1:200 dilution for VEGF-C staining. Mouse anti-human COX-2 antibody (Invitrogen) at 1:100 dilution was used for COX-2 staining. Rectal carcinoma tissue was used as positive control for VEGF-C and urinary bladder carcinomas tissue was used as positive control for COX-2. Rabbit polyclonal antihuman antibody, sc654 (Santa Cruz) was used at 1:750 dilution for eNOS staining. Lung carcinoma tissue was used as positive control. Mouse monoclonal antihuman MIB-1 antibody was used for staining Ki-67 at a 1:100 dilution.

**Fig. 1:**
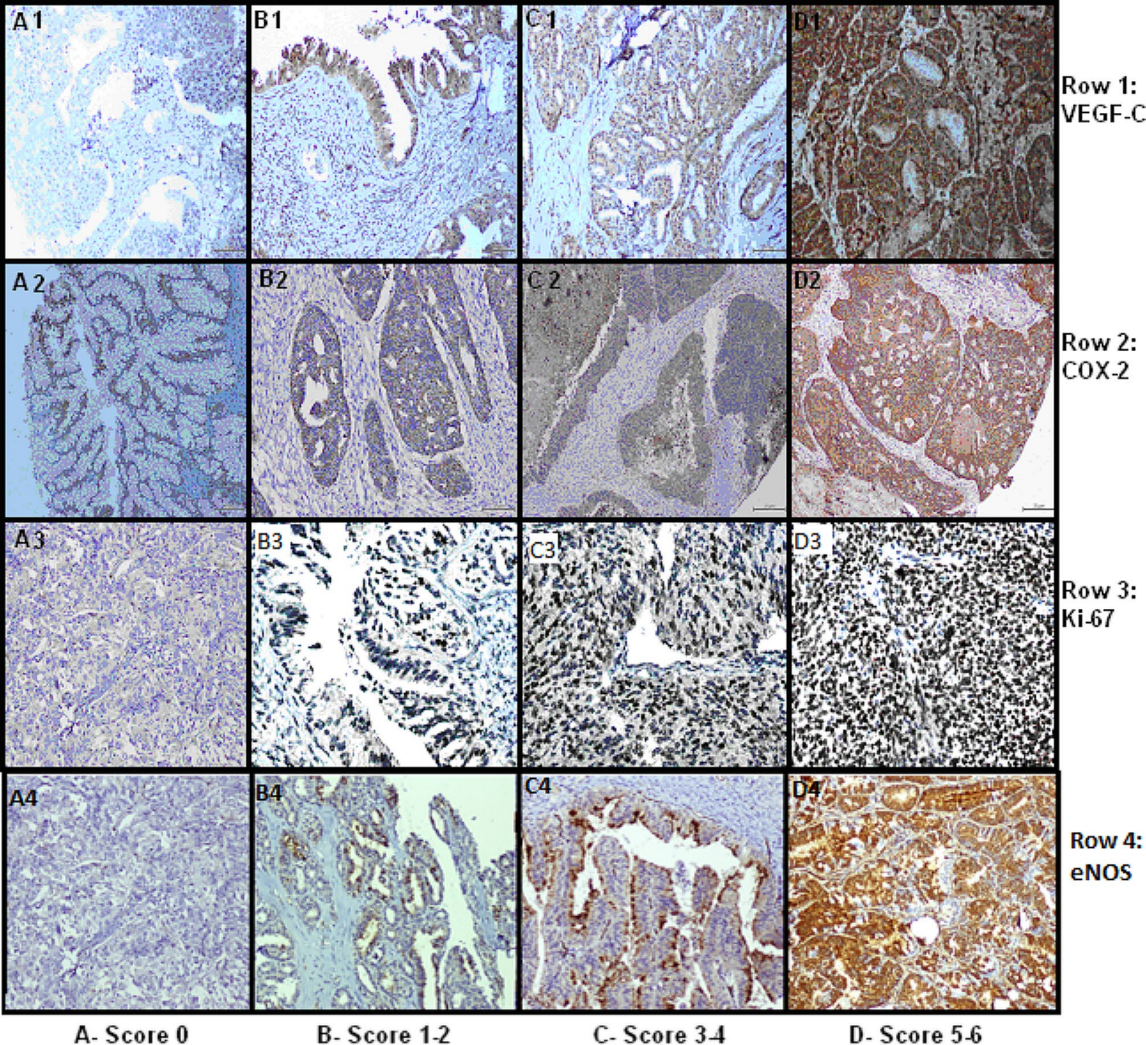
IHC staining of eNOS, VEGF-C, COX-2 and Ki-67

The IHC scoring was based on the presence of staining, intensity and percentage of tumour cells exhibiting staining. The scoring for the immunohistochemical staining is given in Table 2. Following the Allred and Quick scoring method, the score for each factor (intensity and percentage cells score) were then added to arrive at a consensual score depicting the staining strength. The final score was classified on a scale of 1–6 with 1–2 being mild staining, 3–4 being moderate staining and 5–6 being strong staining. Ki-67 showed nuclear staining and expressed as percentage of cells stained. Fig. 1 shows the immunohistochemical staining of VEGF-C, COX-2 and Ki-67 with graded scoring. (For Ki-67, it is only the percentage cells-stained column to be assessed).

**Table 2:** IHC scoring for VEGF-C, COX-2 and eNOS

Intensity	Percentage	Staining	Score
---	< 1	No	0
Weak	1–10%	+	1
Moderate	10–50%	++	2
Strong	> 50%	+++	3

### Statistical analysis

The Chi square and Fischer’s exact test were applied to examine the impact of markers with disease status and other clinicopathological factors. Non-parametric Spearman’s rank correlation test was applied to define the association between each test factor. All statistical analyses were done using SPSS version 21. *P* values were two-tailed. All differences were considered significant for *P* values < 0.05.

## Results

**Fig. 2:**
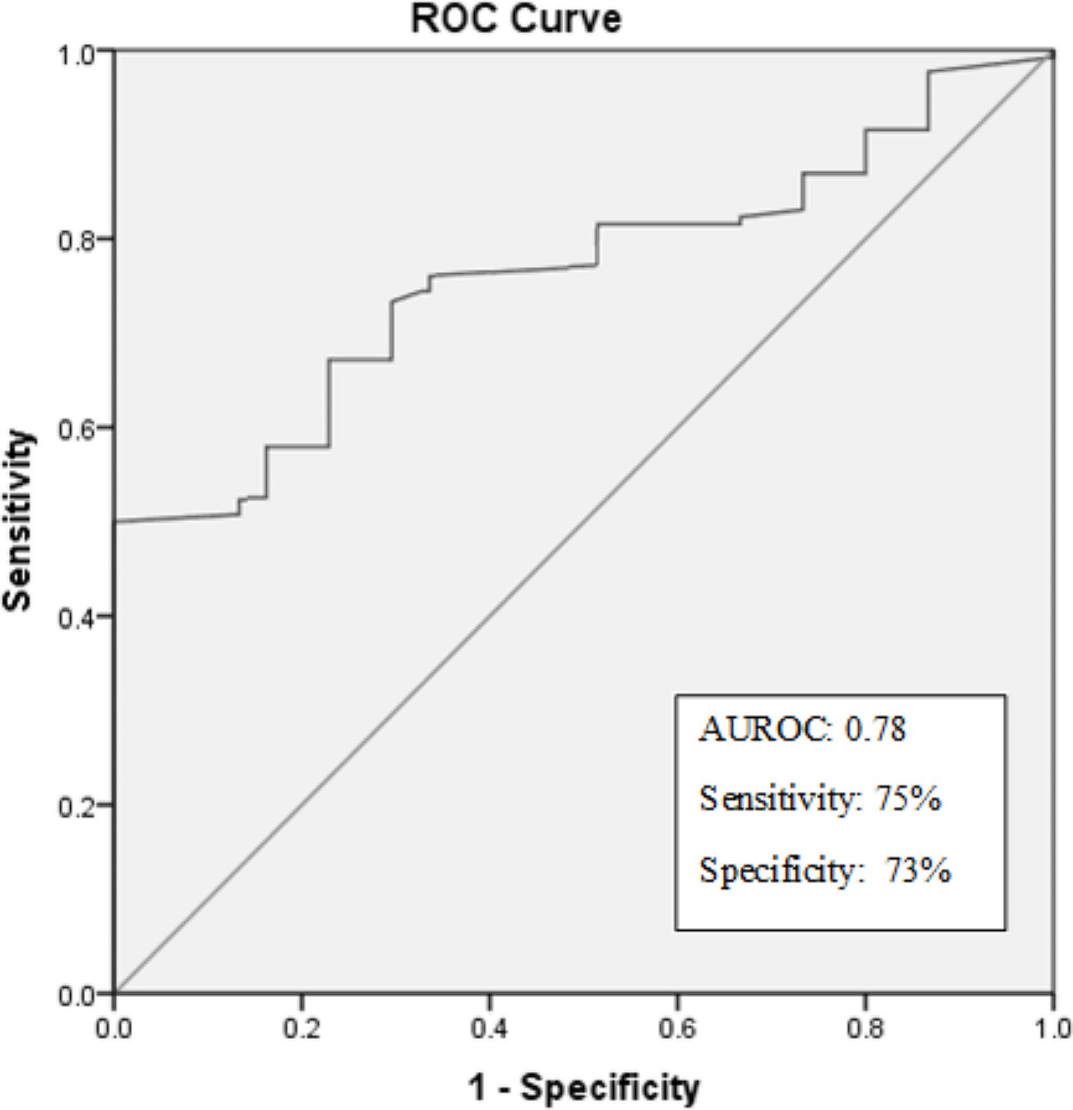
ROC analysis of VEGF-C plasma levels

**Fig. 3:**
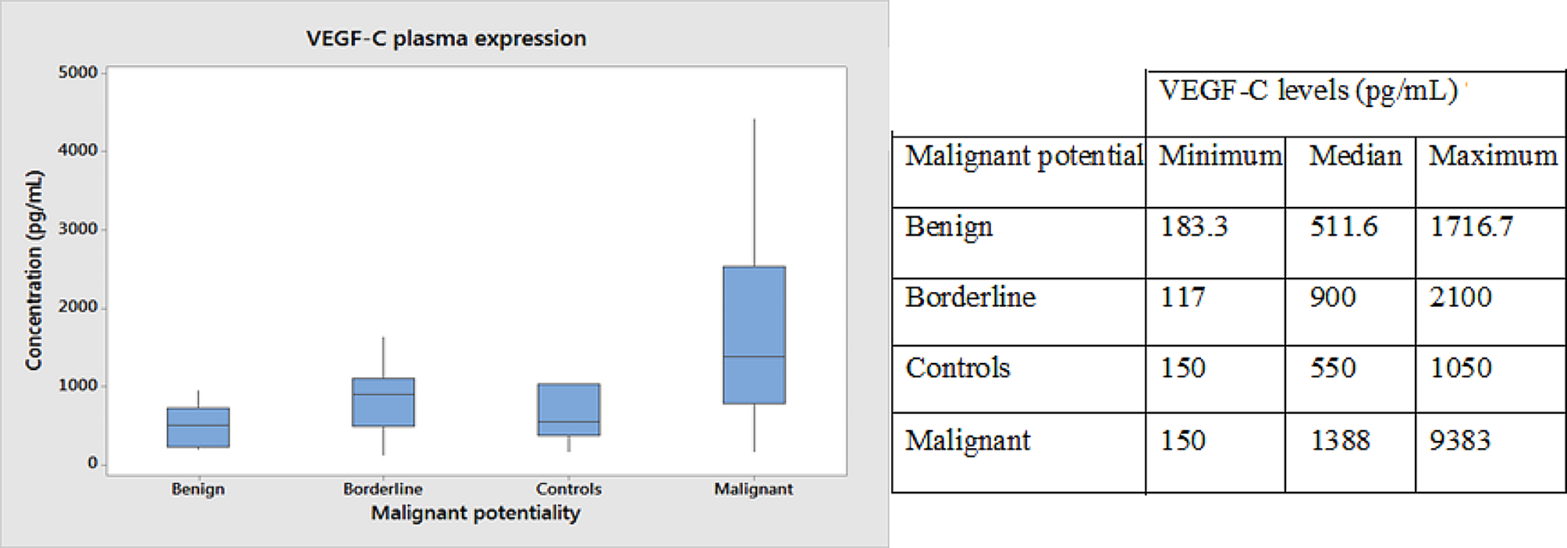
VEGF-C plasma distribution

The plasma levels of VEGF-C were highly up-regulated in the malignant subset with no significant difference in either benign or borderline cases. The mean concentration in malignant, borderline, benign and control samples were 2031 pg/mL, 905pg/mL, 559pg/mL, and 639pg/mL respectively. The increase in the plasma VEGF-C levels of borderline cases was not statistically significant. The average value of plasma VEGF-C was 2083pg/mL in the high-grade malignancies and only 1636pg/mL in low grade malignancies. The difference was statistically significant with a *p*-value of < 0.001. An ROC curve analysis set the cut-off value at 900pg/mL as a fair indicator of malignancy. Higher plasma expression correlated with recurrence in patients. The ROC graph is shown in Fig. 2 and the plasma distribution of VEGF-C is shown in Fig. 3.

The tissue expression of VEGF-C was up-regulated across all subsets of the EOC cases. Yet, the plasma VEGF-C level was a better indicator of aggressive disease and recurrence. Tissue expression was observed to be very strong in high-stage and high-grade disease but did not correlate with disease aggression or recurrence. An interesting observation was that 64% of malignant cases exhibited nuclear staining of VEGF-C and it correlated positively with increased plasma levels of VEGF-C. Nuclear staining of VEGF-C was observed in those cases that were diagnosed with high stage/grade disease and eventually presented with aggressive disease, recurrence, and mortality. Could the nuclear presence of VEGF-C be a marker for aggressive disease or worse prognosis? This possibility needs more clinical or explorative studies with a larger number of patients to ascertain.

COX-2 tissue expression associated strongly with malignancy (*p*-value of < 0.001), and the expression was just moderately up-regulated in borderline and benign cases. COX-2 and Ki-67 expression were found to be concurrently up-regulated.

**Fig. 4:**
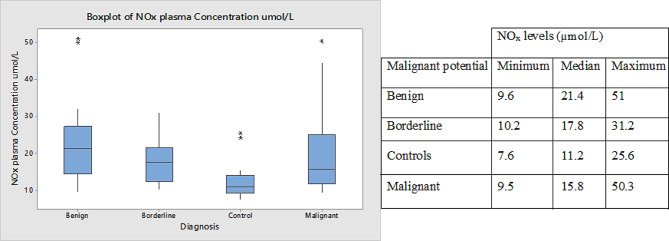
NOx plasma distribution

**Fig. 5:**
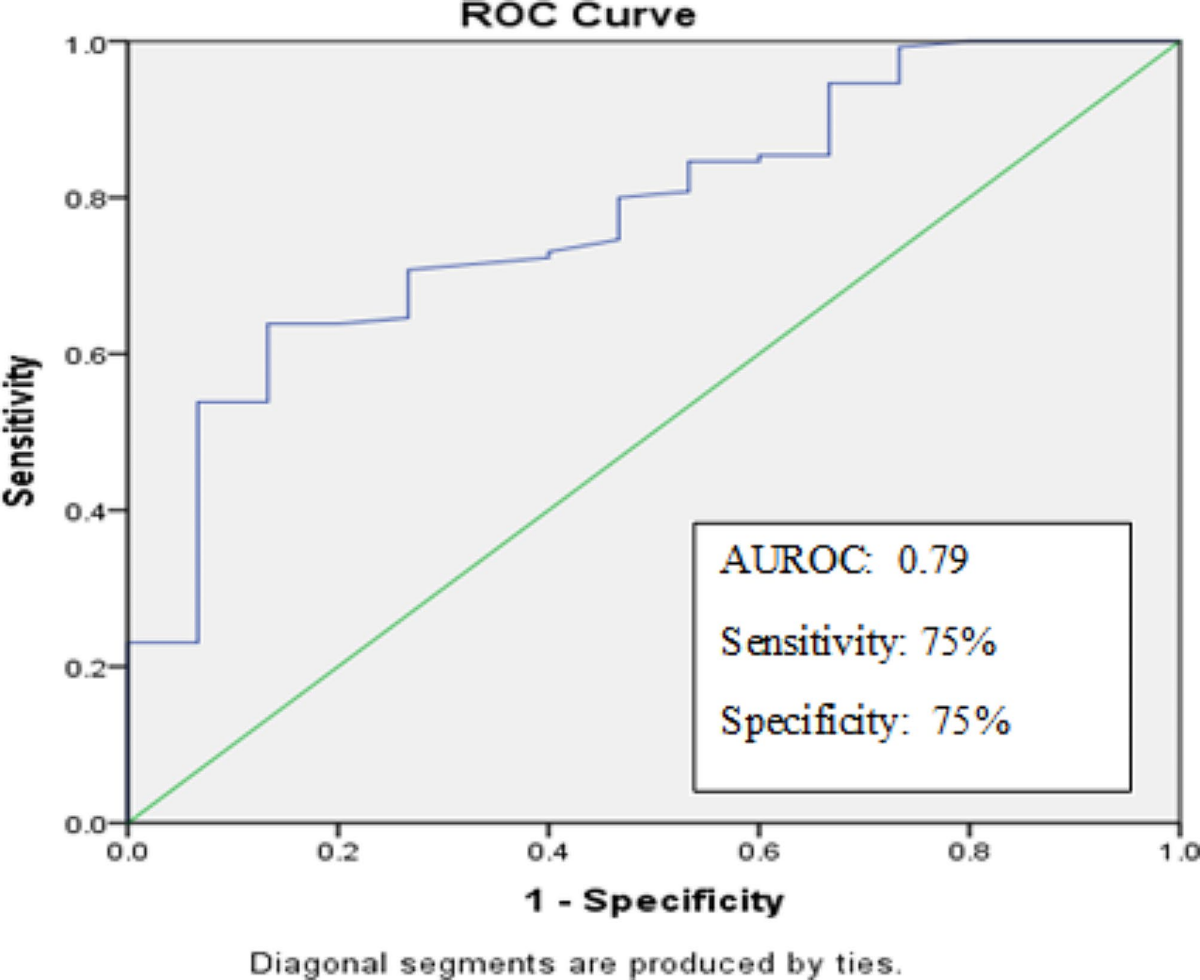
ROC graph for NOx plasma levels

Fig. 4 shows the plasma distribution for total NOx and Fig. 5 shows the ROC graph. The mean NOx levels were 12.95 ± 5.3 µmol/L, 19.71 ± 9.8 µmol/L, 22.88 ± 11.9 µmol/L, and 17.94 ± 6.4 µmol/L in the control, malignant, benign, and borderline subsets respectively. Although the plasma nitric oxide levels were upregulated in EOC, this difference was not statistically significant.

eNOS tissue expression significantly associated with malignancy (*P* value 0.048). Borderline and benign samples showed mild or no eNOS staining. It was observed that most well differentiated tumours showed higher expression of eNOS, especially the mucinous sub-type, although this was not statistically significant.

The plasma VEGF-C, tissue COX-2 and tissue Ki-67 exhibited highly significant positive correlations with the FIGO stage, grade, malignancy, bilateral affliction, CA-125 levels and ascites presence. Plasma VEGF-C and tissue COX-2 were highly promising predictors of recurrence and mortality. Table 3 shows the associations between the various test factors and the clinicopathological parameters in brief. A detailed summary of the same in given in Supplementary Table 1 that includes the chi-square values, number of cases and corresponding values of significance.

**Table 3:** Association of test factors with clinicopathological factors

Test Factors/Clinico-pathological factors	VEGF-C plasma levels	VEGF-C tissue expression	COX-2 tissue expression	eNOS tissue expression	NOx plasma levels	Ki-67 tissue expression
FIGO stage (I, II Vs III IV)	**< 0.001**	0.251	**< 0.001**	0.175	0.271	**0.006**
Grade (low Vs high)	**< 0.001**	0.209	**< 0.001**	0.329	0.638	**0.034**
Malignancy (Malignant Vs benign/controls)	**< 0.001**	0.201	**< 0.001**	0.060	0.361	**< 0.001**
Histopathology (Serous Vs Others)	0.268	0.541	**< 0.001**	0.574	0.481	0.601
Bilateral-affliction (Presence Vs Absence)	**0.001**	0.219	**0.012**	0.624	0.059	**< 0.001**
Pre-opCA125 levels (Elevated Vs Normal)	**0.004**	0.875	**< 0.001**	0.879	0.502	**< 0.001**
Ascites (Presence Vs Absence)	**0.037**	0.758	**0.006**	0.537	0.086	**< 0.001**
Residual disease (< 1 Vs > 1)	0.174	0.862	0.142	0.710	0.860	**0.002**
Recurrence (Presence Vs Absence)	**< 0.001**	0.560	**< 0.001**	0.797	0.814	0.276

Elevated plasma NOx levels associated statistically with bilateral ovarian affliction, CA-125 levels and ascites production (*P* values < 0.05). eNOS tissue expression did not associate with any of the other clinico-pathological parameters either. Spearman’s correlation coefficient analysis revealed no correlation between the plasma NOx levels and eNOS tissue expression, suggesting that eNOS might not be a major perpetrator in EOCs. Table 4 shows the spearman’s correlation coefficient between the test factors.

**Table 4:** Correlation between test factors

Spearman’s Corelation Coefficient				
Test factors	VEGF-C plasma	eNOS tissue	COX-2 tissue	NOx plasma
VEGF-C tissue	0.237**	0.172	0.038	-0.212*
Ki-67 tissue	0.270*	0.229	0.570**	-0.171

** Correlation is significant at the 0.01 level (2-tailed)

* Correlation is significant at the 0.05 level (2-tailed)

## Discussion

VEGF-C plasma levels significantly correlated with disease aggression and recurrence (*p*-value < 0.001). It was observed that the patient sub-set with highly elevated levels of VEGF-C in their plasma, at their initial presentation, came back with recurrence (post-surgery) much faster than the sub-set of patients with mildly elevated VEGF-C plasma levels. Up-regulated tissue expression was observed in all subsets of EOC cases but was not a specific marker for malignancy. Thus, a case with an increase in both tissue expression and plasma levels would be a candidate for very aggressive disease or poor prognosis. Patients with only increased tissue expression and no plasma expression can probably be marked for mild disease status. Plasma VEGF-C, is hence, a significant prognostic marker.

An interesting finding in our study is the nuclear localization of VEGF-C in ∼ 50% of the cases. It was also observed that 100% of the samples that exhibited nuclear staining were high grade and showed elevated plasma VEGF-C levels. This correlated with disease aggression and recurrence. The nuclear localization of VEGF-C seems to be a later molecular event that happens and seems to cause a subsequent increase in secreted levels of VEGF-C. There have been a few reports that have elucidated the nuclear localization of VEGF-A and VEGF-D under hypoxic conditions that eventually cause an up-regulatory modulation of the self-molecule or of other angiogenic molecules [20, 21]. We have not been able to find any published reports on the nuclear localization of VEGF-C in a carcinoma. This is a novel finding that warrants further studies to elucidate the exact molecular phenomenon involved. At this juncture, it is not clear if the nuclear staining of VEGF-C is a specific prognostic marker, but we postulate that it is potentially a specific marker for late-stage disease, distant metastasis, and malignancy even at initial presentation or clinical assessment of a patient.

In the event of carcinogenesis and ascitic fluid build-up, it is quite expected for tissue VEGF-C to be up-regulated since lymphatic drain is a common mechanism of the body to deal with fluid build-up. Furthermore, ovarian tissue has been reported to constitutively express VEGF-C, owing to the constant process of wear-tear, and consequently reflect lymphatic processes for repair and maintenance. Yet, this does not de-facto cause vascular leakage or tumorigenesis. But, when there are multiple factors like VEGF-A, COX-2 and NO involved, the expression profile changes significantly. All these were well up-regulated in our study and were shown to induce VEGF-C expression in various studies. The impact of multiple factors up regulating its expression, is obvious in our study where a strong expression was documented and that correlated significantly with recurrence and mortality. This leads to the postulate that incumbent VEGF-C tissue expression does not cause much harm but the concomitant cumulative stimulation from multiple factors causes a mob effect of circulating rogue VEGF-Cs that create a tip in the internal balance towards metastatic proliferation and hence poor prognosis.

eNOS has a multi-faceted role in carcinogenesis, especially EOCs. It has not been explored and there is dearth in data regarding eNOS tissue expression status and NOx plasma levels in EOCs. VEGF-A is known to induce eNOS via PLC/IP_3_ and at the same time suppress eNOS via the PKC mediated pathway. Gelinas DS et al. in their study proposed that VEGF downstream signalling activated PLC→IP_3_→Ca^2+^ influx→eNOS activation. eNOS could also be activated via the PI_3_K/Akt nexus. They also showed that VEGF-A downstream signalling induced the p38 MAPK and p42/44 MAPK [22]. This p38 MAPK was shown to downregulate eNOS expression by Xing et al. [23].

Our precedent work showed VEGF-A is highly over-expressed in EOCs. Hence, it was surprising to see in the current study, that only a moderate up-regulation of eNOS was observed in the malignant subset of cases and was statistically not significant. We can postulate that VEGF-A downstream signalling maintains a modulatory stance on eNOS expression, possibly via the attenuating action of p38 MAPK expression despite other factors that induce eNOS under hypoxic conditions.

eNOS is produces only nanomolar concentrations of NO in contrast to iNOS, that produces micromolar concentrations of NO [24]. NO is pro-tumoral at lower (nanomolar) concentrations while it is tumoricidal at higher (micromolar) concentrations. This is the reason why iNOS produces a NO burst that is used for bactericidal purpose by macrophages. In our study we found diffusely elevated levels of total nitric oxide in patient plasma suggesting that there is no marked induction of NO in EOCs. This affirms to the fact that; it is low concentrations of NO that promote angiogenesis but not higher concentrations. Nitrites, nitrates, S-nitroso thiols, and nitrosamines are NO metabolites and mediators of it effects [25]. The effect of NO depends on its concentration, type of cell exposed to NO, redox state of the cell, duration of exposure, etc. It has also been reported that NO circulates in mammalian plasma primarily as an S-nitroso adduct of serum albumin [26]. In EOCs, there is considerable loss in albumin in patients. Hence there could be a dearth for the primary source of storage for NO mediators. This could probably explain why there is no markedly elevated levels of plasma NO.

NO modulation has become one of the obvious targets for treatment of cancers. Although, in our study we found no association with malignancy. Yet, it predictably associated with ascites levels and CA125 levels. On the other hand, eNOS associated significantly only with malignancy and no other clinicopathological factors.

The complexity in EOCs and difficulty in diagnosis and prognosis are evident. The prognosis remains poor for advanced stage diseases. Although eNOS is upregulated, it fails as a prognostic marker since it did not associate with disease free survival or recurrence. NO levels on the other hand seemed to predict the extent of ascites production and this could in turn be indicative of poor prognosis. Massive ascites often renders EOCs aggressive and present complex symptoms in patients. Malignant ascites is also known to increase chances of metastasis and rapid secondary tumour formation. This is a first such study enquiring into the eNOS tissue expression and plasma NOx levels in EOC patient samples and provides details regarding the in vivo tumour microenvironment.

Our study has yielded some significant findings in the turf of epithelial ovarian cancers, especially in the scenario of a pan-Indian population (Asian race). In a nutshell, from a panel of VEGF-C, COX-2, Ki-67, eNOS, NOx, the VEGF-C plasma levels and nuclear staining of VEGF-C were promising markers for aggressive disease. While Ki-67, tissue COX-2 and VEGF-C plasma levels were formidable and independent markers for poor prognosis, only the latter two strongly predicted recurrence and hence can effectively stratify patients for a customised mode of treatment.

### Electronic supplementary material

Below is the link to the electronic supplementary material.

Supplementary Material 1

## Data Availability

All data generated and analysed during this study are included in this published article.
