# Supplementary material for: Pre-operative plasma VEGF-C levels portend recurrence in epithelial ovarian cancer patients and is a bankable prognostic marker even in the initial assessment of a patient
Source: J Ovarian Res. 2024 Apr 9;17:77. doi: 10.1186/s13048-024-01398-0 (PMC11003002; doi:10.1186/s13048-024-01398-0)
Supplement: Supplementary file 1 — Supplementary Material 1 [file 13048_2024_1398_MOESM1_ESM.docx]

**Supplementary Table 1: Association of test factors with clinicopathological factors**

| Clinicopathological factors | VEGF-C plasma levels | VEGF-C tissue expression | COX-2 tissue expression | eNOS tissue expression | NOx plasma levels | Ki-67 tissue expression |
| --- | --- | --- | --- | --- | --- | --- |
| **late stage** | **<0.001** | **0.251** | **<0.001** | **0.175** | **0.271** | **0.006** |
| Pearson chi-square value | 30.72 | 5.38 | 24.21 | 13.25 | 3.75 | 7.42 |
| No: of cases | 39/59 | 30/59 | 46/59 | 15/52 | 42/59 | 28/28 |
| P value | 0.00 | 0.251 | 0.00 | 0.175 | 0.271 | 0.006 |
| **High Grade** | **<0.001** | **0.209** | **<0.001** | **0.329** | **0.638** | **0.034** |
| Pearson chi-square value | 24.17 | 4.54 | 32.48 | 6.82 | 0.974 | 4.49 |
| No: of cases | 37/56 | 29/56 | 46/56 | 14/50 | 41/56 | 30/31 |
| P value | 0.00 | 0.209 | 0.00 | 0.329 | 0.638 | 0.034 |
| **Malignancy** | **<0.001** | **0.201** | **<0.001** | **0.060** | **0.361** | **<0.001** |
| Pearson chi-square value | 21.44 | 3.212 | 32.34 | 12.18 | 1.99 | 33.95 |
| No: of cases | 63/95 | 52/95 | 73/95 | 26/87 | 71/130 | 41/60 |
| P value | 0.00 | 0.201 | 0.00 | 0.06 | 0.361 | 0.000 |
| **Histopathology** | **0.268** | **0.541** | **<0.001** | **0.574** | **0.481** | **0.601** |
| Pearson chi-square value | 9.96 | 3.102 | 22.86 | 9.76 | 3.56 | 0.273 |
| No: of cases | 32/74 | 41/74 | 49/74 | 18/66 | 54/74 | 24/35 |
| P value | 0.268 | 0.541 | 0.00 | 0.574 | 0.481 | 0.601 |
| **Bilateral affliction** | **0.001** | **0.219** | **0.012** | **0.624** | **0.059** | **<0.001** |
| Pearson chi-square value | 13.23 | 1.97 | 6.65 | 2.24 | 3.96 | 17.51 |
| No: of cases | 43/59 | 36/59 | 47/59 | 19/59 | 45/59 | 25/26 |
| P value | .001 | 0.219 | 0.012 | 0.624 | 0.059 | 0.00 |
| **Pre-op CA125 levels** | **0.004** | **0.875** | **<0.001** | **0.879** | **0.502** | **<0.001** |
| Pearson chi-square value | 18.83 | 0.69 | 22.05 | 4.76 | 2.4 | 23.48 |
| No: of cases | 61/90 | 47/90 | 68/90 | 22/83 | 76/90 | 42/52 |
| P value | 0.004 | 0.875 | 0.00 | 0.879 | 0.502 | 0.00 |
| **Positive Ascites** | **0.037** | **0.758** | **0.006** | **0.537** | **0.086** | **<0.001** |
| Pearson chi-square value | 10.20 | 0.55 | 10.23 | 5.198 | 4.89 | 19.29 |
| No: of cases | 55/93 | 46/93 | 64/93 | 25/81 | 71/93 | 37/44 |
| P value | 0.037 | 0.75 | 0.006 | 0.5198 | 0.086 | 0.00 |
| **Residual disease** | **0.174** | **0.862** | **0.142** | **0.710** | **0.860** | **0.002** |
| Pearson chi-square value | 8.99 | 0.75 | 5.44 | 6.04 | 0.78 | 9.77 |
| No: of cases | 15/51 | 10/51 | 16/51 | 16/48 | 16/51 | 14/14 |
| P value | 0.174 | 0.862 | 0.142 | 0.710 | 0.86 | 0.002 |
| **Recurrence (<3 years)** | **<0.001** | **0.560** | **<0.001** | **0.797** | **0.814** | **0.276** |
| Pearson chi-square value | 21.75 | 1.2 | 19.75 | 1.23 | 0.72 | 1.19 |
| No: of cases | 23/35 | 19/35 | 27/35 | 10/34 | 26/35 | 20/21 |
| P value | 0.00 | 0.560 | 0.00 | 0.797 | 0.814 | 0.276 |
